# Buffalo Immune Competence Under Infectious and Non-Infectious Stressors

**DOI:** 10.3390/ani15020163

**Published:** 2025-01-10

**Authors:** Maria Giovanna Ciliberti, Antonella Santillo, Mariangela Caroprese, Marzia Albenzio

**Affiliations:** Department of Agriculture, Food, Natural Resources, and Engineering (DAFNE), University of Foggia, 71122 Foggia, Italy; maria.ciliberti@unifg.it (M.G.C.); mariangela.caroprese@unifg.it (M.C.); marzia.albenzio@unifg.it (M.A.)

**Keywords:** immune response, cytokines, biomarkers

## Abstract

Immune competence is a critical aspect of protecting animals from the negative consequences of disease. The activation of the immune response from inflammation is part of adaptive homeostasis that serves to eliminate danger, induce tissue repair, and restore tissue homeostasis. Therefore, the main goal for the organism is to control both the induction and suppression of inflammation and resist the onset of disease. In this condition, modulators of inflammatory responses are produced, including small proteins called cytokines, which exert a pro- or anti-inflammatory action in a context-dependent manner. Indeed, the cytokine profile could be considered a useful biomarker to determine the pathophysiology of certain diseases, such as mastitis, endometritis, change-induced heat stress, and zoonoses. Recently, buffalo breeding has attracted the interest of the research communities due to their high resilience; however, little is known about the immune mechanism activated under specific stressors. This review describes the complex immune competence of the buffalo in the presence of the most common infectious and non-infectious stressors. In addition, a brief description of methods for early diagnosis of disease using cytokine quantification will be introduced.

## Simple Summary

In the last decade, buffalo farming has been attracting the interest of the research communities because of its high resilience in the current climate change context. Over their lifetime, animals face a range of infectious and non-infectious stressors on which successful recovery of their optimal performance may depend on different psychological conditions. Enabling both innate and adaptive immune responses, through the secretion of pro- and anti-inflammatory cytokines, plays a key role in the onset or progression of disease. This review describes the complex immune competence of the buffalo in the presence of the most prevalent infectious stressors, including mastitis, endometritis, and non-infectious stressors such as climate change-induced heat stress.

## 1. Introduction

Currently, infectious diseases in buffaloes (*Bubalus bubalis*) are managed according to the method validated for cattle (*Bos taurus*); however, to adapt cattle-based strategies, it is crucial to understand the buffalo immune system and the variables involved in disease susceptibility and resistance. Furthermore, an in-depth understanding of the mechanisms of thermal adaptation and related physiology of immunity is critical to studying new mitigation strategies to cope with the climate change condition, which is one of the most well-known abiotic stressors for farm animals, including buffalo [1]. In this context, the complex nature of the relationship between animal welfare and an animal’s immune status is worthy of attention. Indeed, the high metabolic cost of mounting an effective immune response could profoundly affect animal performance and welfare [2]. Available welfare assessment protocols, based on the assessment of individual herds, may include parameters that directly or indirectly reflect their health; however, data on health routinely recorded may not be immediately available. Therefore, any respiratory, enteric, and reproductive problems, including prolapse and mastitis at an early stage, should be recorded with the aim of improving animal welfare conditions in farms and avoiding high mortality and culling rates due to disease and accidents [3].

No previous study has summarized the immune competence of buffalo in the presence of different infectious and non-infectious stressors and very little is known about the complex mechanisms specifically activated by the buffalo immune system. The present review aims to analyze the main immune responses activated by buffaloes under different infectious (mastitis, endometritis, and zoonoses) and non-infectious (climate change-induced heat stress) stressors. Furthermore, an overview of the immune systems and the role of the main immune molecules (cytokines and miRNAs) studied in buffaloes is presented.

## 2. Overview of the Immune System

Adaptive homeostasis is defined as the transient change in biological response in the face of exposure and or cessation to such molecules or events [4]. This definition describes the plasticity of cells or organisms to adapt to any transient changes better than the term homeostasis. Stressors can be both internal to the organism or environmental, along with heat stress, cold stress, exercise, oxidative stress, food deprivation, hypoxic or anoxic stress, chemical toxins, heavy metals, mechanical stress, salt, alcohol, osmolarity, and even emotional and psychological stresses, as well as others [5,6,7]. The immune system includes a variety of biological components and processes that aim to protect animals from the consequences of disease [8]. The activation of the immune response by inflammation after recognition of any condition perceived as potentially dangerous to the host is part of adaptive homeostasis, which aims at the removal of the danger, induction of tissue repair, and restoration of tissue homeostasis. Based on this last concept, the main goal for the organism is to control both the induction and suppression of inflammation [9] and resist the establishment of disease. Two main compartments are characteristic of the immune system: the innate and the adaptive systems, which actively cooperate with each other. While innate immunity is fundamental during the onset of pathogen invasion or any tissue insult in the first seconds and minutes, adaptive immunity takes several days to organize a response; although innate immunity is intended for a broad scope, the second one is customized and more specific. Innate immune defense is composed of the physical barrier of the skin and mucous epithelia, immune cells (macrophages, neutrophils, and natural killer cells), non-immune cells (epithelial and endothelial cells), and soluble mediators (cytokines, eicosanoids, and acute phase proteins). Neutrophils, macrophages, and dendritic cells discriminate pathogens and self by utilizing signals from Pattern Recognition Receptors (PRRs) among which are Toll-like receptors (TLRs) [10]. Pattern recognition receptors are able to recognize a range of microbial factors associated with infectious pathogens and can be expressed on cell surfaces, secreted, or expressed intracellularly [11,12]. The first PRR identified was Toll in *Drosophila melanogaster*, and the toll-like receptors (TLR) are now among the most widely studied PRRs in mammals [13]. The innate immune system can recognize specific pathogens through associated molecular patterns (PAMPs) as TLR signals. Moreover, TLRs can induce the specific cytokine network, causing a certain degree of specificity to the innate immune system and consequently influencing the nature of the adaptive immune response. Examples of PRR found in both immune and nonimmune cells that can differentiate a range of PAMP are a cluster of differentiation (CD)14, nucleotide-binding oligomerization domains, and the family of TLR, including TLR2, TLR4, TLR5, and TLR9. After binding to their ligand, PRR can begin intracellular signaling cascades that result in the initiation of innate immune responses or can facilitate antimicrobial activity directly [12]. The acute phase response is activated by the release of inflammatory cytokines, especially interleukin (IL)-1, IL-6, and tumor necrosis factor (TNF)-α from the macrophages or blood monocytes at the site of inflammatory lesions or infections [14]. Cytokines are small proteins released by cells, which have a specific effect on the interactions and communications between cells. Chemokines are a broad class of small proteins that signal through G protein-coupled heptahelical chemokine receptors on cell surfaces. Furthermore, cytokines can provide a polarizing signal, which is one required for the activation of T cells by antigen-presenting cells. Among the cytokines, IL-12 activates a T helper (Th)1-type response, which generates a cell-mediated inflammatory response to intracellular microbes [15], whereas IL-4 and IL-10 drive mainly Th2-type responses [16], which generate antibody-mediated inflammatory responses toward extracellular pathogens. In relation to the type of infectious agent, TLRs cause skewing toward the production of either pro-inflammatory Th1 type cytokines (IL-12, interferon-IFN-γ, and TNF-α) or Th2 type cytokines (IL-4 and IL-10), which promote an anti-inflammatory response [17]. Inteleukin-10 is considered a crossroads between the innate and the adaptive immune responses via activating the recruitment of monocytes and macrophages, mediated by the induction of chemokine-4 and some scavenger receptors, responsible for antibody-dependent cell-mediated cytotoxicity and phagocytosis of opsonized particles [18,19]. Inteleukin-10, together with IL-4, plays an important role in the regulation of Th2 cells and in the development of humoral immune responses by stimulating the growth of activated T cells [20]. The main biological function of IL-1–type cytokines (IL-1α and IL-1β) is to control proinflammatory reactions in response to tissue injury by PAMPs, such as bacterial or viral products or DAMPs, among which are uric acid crystals or adenosine 5′-triphosphate [21]. Interleukin-6 type cytokines are responsible for the activation of target genes involved in differentiation, survival, apoptosis, and proliferation and can manifest in relation to both pro-inflammatory effects (for example, in acute innate responses) and anti-inflammatory activities, which are essential for the resolution of inflammation [22]. Tumor necrosis factor-α (also known as cachectin) is a strong pro-inflammatory cytokine that shows an important role in the immune system during inflammation, cell proliferation, differentiation, and apoptosis [23], and its production is activated immediately after recognition of an invading pathogen by TLRs [24], inducing a cascade of signals, which increase vascular permeability and promote the passage of macrophages and neutrophils to the site of infection [25].

Cytokines can also act as mediators between the local site of injury and the hepatocytes (liver) to produce and release the acute phase proteins (APP) that play a central role in the systemic reaction to inflammation. Indeed, APP has an active role in the opsonization of several pathogens, the scavenging of potentially toxic substances, and the regulation of different stages of inflammation. During the acute phase response, the serum concentration of the APP changes dramatically. The APP can be classified according to their concentration in positive APP if they increase or negative APP if they decrease [26]. Interestingly, some APPs are downregulated during the acute phase in serum, while extrahepatic tissues are upregulated, such as albumin, which is a positive APP in the mammary gland during mastitis [27]. In ruminants, the serum amyloid A (SAA), haptoglobin (Hp), lipopolysaccharide-binding protein (LBP), and α1-acid glycoprotein (AGP) are regarded as the four major APPs that can have a binding protein effect and regulate innate immunity reactions [28]. Another class of molecules that are essential components of the innate immune system with immunomodulatory action are the antimicrobial peptides (AMPs). Among them, defensins, cathelicidins, and hepcidin are the mostly studied in buffalo, as reviewed by Chanu, et al. [29], and mainly considered as the first line of defense against foreign invaders, neutralizing the invading pathogens with a broad-spectrum activity against both gram-positive and gram-negative bacteria.

## 3. Mastitis Infection

Mastitis is mainly caused by bacterial infection of the mammary gland, which determines dramatic economic losses in buffalos’ dairies due to negative influence on the milk quality and yields [30], treatment costs, replacement animal costs, and reduced ability to market dairy products [31]. A broad range of both gram-positive and gram-negative pathogens can cause mastitis, establishing the severity of infection by the expression of bacterial virulence factors. Moreover, the susceptibility and the extent of disease severity in dairy animals are also influenced by host-related factors, among which are nutrition, genetics, oxidative stress, environmental stressors, and the physiological transition of the mammary gland from the dry period to lactation [31]. Mastitis is characterized by pain and local inflammation due to the presence of proinflammatory cells that secrete prostaglandin, histamine, serotonin, and interleukins, which, in turn, trigger the vasodilatation of the mammary capillaries, thus increasing the temperature of the udder [32].

In buffaloes, the pathogens most frequently involved in mastitis are coagulase-negative *Staphylococci* [30], *Staphylococcus* spp., *Streptococcus* spp., and *Corynebacterium* sp. [33]. The coliform bacteria (*Klebsiella pneumoniae* and *Escherichia coli*) are the most widespread pathogens, followed by *Staphylococcus aureus*, *Streptococcus uberis*, and *Streptococcus agalactia* [34]. Basically, the determination of milk somatic cell count (SCC) is used to monitor udder health and diagnose mastitis [30]. Previous studies in buffaloes indicate that quarters producing bacteria-negative milk with SCC < 200 × 10^3^ cells/mL are considered free from intra-mammary infections (IMIs) or healthy [30,35,36]. On the contrary, buffaloes producing milk with SCC upon the threshold of 200 × 10^3^ and with positive bacteriological cultures are defined as suffering from subclinical mastitis (SCM). In cases of clinical mastitis (CM), obvious signs of disease involving the appearance of the milk or udder are evident [30,35,36]. In the condition of SCM, there are no clinical symptoms or visible changes in milk or udder appearance, even though the immune system responds by sending defense cells to fight the pathogen. Non-specific immunity is activated during the early stage of mastitis, promoting the rapid elimination of most pathogens [37] mediated by TLRs, which are expressed on mammary epithelial cells and the leukocytes present in milk [38]. After the recognition of pathogens, several effector molecules are released and activated [39], including antimicrobial peptides and cytokines. In the mammary gland environment, the immunomodulatory capacity of the cytokine network is very complex [40]. Moreover, the critical role of certain cytokines in the pathophysiology of mastitis has been demonstrated [41]. Indeed, when the udder tissue becomes infected with *S. aureus*, causing inflammation, the number of Th1 and Th2 type cells increases, mediated by the pro-inflammatory cytokines regulated by IL-10 [42]. During *E. coli* infection, the release of IL-10 helps in controlling excessive levels of pro-inflammatory cytokines and in preventing excessive tissue damage and hyperimmune reactions [43]. A different outcome has been demonstrated among Crossbred cattle, Tharparkar cattle, and Murrah buffalo of peripheral blood mononuclear cells (PBMCs) on IL-10 cytokine release in response to in vitro antigen stimulation (Peptidoglycan + Lipoteichoic acid as a representative of *S. aureus* and lipopolysaccharides (LPS) as a representative of *E. coli* antigens), which indicates that breed/species suffering from the same type of infection have a different susceptible or tolerance/resistant pattern [44]. In a study by Tanamati et al. [45], Murrah buffaloes suffering subclinical mastitis experience an increase in mRNA expression of the *TLR-2* and *TLR-4* genes with a concomitant higher mRNA expression of *TNF-α*, *IL-1β*, and *IL-8* genes in somatic cells (SCs), which have been registered. Conversely, mRNA expression of lactoferrin (LTF), which downregulates proinflammatory cytokines and prevents the release of inflammatory mediators [46], has not been changed among SCs from mastitis or no mastitis buffaloes [45]. In another study [41], SCs from infected mammary glands have increased amounts of mRNA for IL-1β, IL-6, IL-8, and IL-12B cytokines. In addition, high levels of IL1, IL8, and TNF-α have shown a strong correlation with the most severe clinical symptoms associated with coliform or endotoxin-induced mastitis [47,48,49,50]. Accordingly, a high relative transcript level of TNF-α has been observed in SCs from buffalo with subclinical mastitis followed by IL-1B, IL-6, and IFN-γ. Moreover, Mansour and Zeitoun [51] have demonstrated that high levels of peripheral cytokines such as TNF-α, IL-6, IL-1β, and IFN-γ cause reproductive failure in buffalo cows suffering from either clinical or sub-clinical mastitis. Indeed, this cytokine milieu has been associated with low follicular estradiol 17β production, resulting in suppression of the luteal function, leading to a significant decline in conception and overall disruption to the function of the pre-ovulatory follicle on the day of estrus [51]. Therefore, monitoring cytokines implicated in the regulation of immune responses during infection could be useful in designing a cytokine marker tool in the early diagnosis of mastitis [52].

A recent shotgun proteomic study [53] demonstrated an increase in proteins involved in the innate immune defense functions, including vimentin, cathelicidins, histones, S100 and neutrophil granule proteins, haptoglobin, and lysozyme, which have been highlighted in water buffalo with mastitis caused by *S. aureus*. Among the increased proteins, cathelicidins are mainly expressed in buffalo milk [53]. Cathelicidins are specific antimicrobial proteins released by milk neutrophils and epithelial cells in response to an intramammary infection [54,55] and can be considered a more specific indicator of mastitis when compared to the total somatic cell count (SCC), which also includes other cell types. For this scope, a cathelicidin ELISA specific for bovine [56] and sheep [57] for detecting mastitis was also applied in buffaloes; however, the results are not encouraging so far and need further optimization by improving the species-specific sequences of antibodies used for ELISA [58]. During mastitis, an accumulation in the milk of polymorphonuclear cells (PMNs) is activated to phagocytose and destroy invading pathogens before the establishment of infection [59]. In this process, antimicrobial defensin peptides are realized from the PMN cells [60]. A unique study on buffalo has demonstrated that two β-defensins (LAP and BNBD-2) have been detected in mastitis milk and in buffalo PMN cells [61], concluding that the determination of β-defensin peptides expression can be considered as an approach to the assessment of cellular and molecular responses to cell injury.

Granulocyte-colony stimulating factor 3 (CSF3) is one of the major hematopoietic cytokines [62], which can take an active role in immune responses such as cell proliferation and survival, differentiation of the neutrophil precursor cells in the bone marrow, leukocyte recruitment in inflammatory sites, lymphocytes and antibodies production during inflammations, and tissue lesions and infections, especially those bacterial in origin [63,64,65]. The lactoperoxidase (LPO) provides a defense system against invasive microorganisms with a protective role on the mammary gland and concomitantly on the intestinal tract of newborns against pathogenic microorganisms found in the milk [66]. In order to verify the role of CSF3 and LPO during mastitis, Stella et al. [67] found that buffaloes suffering from mastitis increase *CSF3* gene expression in comparison with healthy animals; on the contrary, the LPO system does not change between healthy and mastitis animals. The results from this study may candidate CSF3 as a biomarker of the mastitis in buffalo. In the study of Restucci et al. [68], buffalo udders affected by chronic sub-clinical mastitis have been characterized by a predominance of interstitial CD8+ lymphocytes (92%) together with tertiary lymphoid structures (TLSs) that can be useful to play a protective role against infectious agents near their entry point [68]. Therefore, CD8+ lymphocytes can be implicated in mastitis infection at the local level by acting as cytotoxic cell scavengers, removing old or damaged secretory cells, and reducing the susceptibility of the gland to infections [69]. Recently, a Water Buffalo Mastitis database (WBMSTDb) (http://webtom.cabgrid.res.in/wbmstdb/ accessed on 8 September 2024) has been built by Jaiswal et al. [70], with the aim of developing a targeted gene panel of mastitis in water buffalo, starting from the one available for bovine. Moreover, Jaiswal et al. [70] have performed the validation of the RNA-Seq library in water buffalo, along with the prediction of biochemical pathways through the protein–protein network analysis. Briefly, the results show that the functional prediction of the key genes associated with mastitis disease involves the immune and inflammatory response; indeed, genes like *CXCL8*, *CD14*, *S100A8*, *CSF2*, *C3*, *Interferon regulatory factor 1 (IRF1)*, *IL1B*, *IL6*, *LPO*, *LBP*, *TLR2*, and *TLR4* are well-known immune and inflammatory responsive genes [71]. Moreover, proteins like complement C4 (C4), BRCA1, TLR4, MBLA, and calcium voltage-gated channel auxiliary subunit alpha2delta 1 (CACNA2D1) have been found during mastitis in buffalo and are also reported as potential biomarkers in livestock mastitis [71].

Recently, the evaluation of the microRNA (miRNAs) profile, a specific class of noncoding RNAs (approximately 22 nucleotides in length), is used as a valuable prognostic biomarker for early subclinical mastitis detection, especially when the California mastitis test could not be conclusive, being characterized by both false positive and negative results. MiRNAs are considered key factors impacting the features and the magnitude of the inflammatory process [72]. Particularly, during pathogen invasion or under injuries, miRNAs activate immune and physiological processes for removing pathogens and maintaining homeostasis. Circulating miRNAs in milk have been used as a novel method of quality control and as a disease biomarker [73]. With this aim, in a recent paper by Jadhav et al. [74], a different upregulation of miR-146a and miR-383 has been observed in buffalo milk between normal, subclinical, and clinical mastitis, and a positive correlation with SCC and subclinical mastitis has been found, suggesting the use of miR-146a and miR-383 as markers for mastitis diagnosis. Another study [75] conducted a comparative analysis of miRNAs, respectively, in buffalo, goat, sheep, and donkey milk and has demonstrated a high expression in buffalo milk of miR-11987, which has been found to have immunomodulatory properties during subclinical mastitis in cows; therefore, its role also needs to be further explored in buffalo mastitis. Table 1 shows the immune biomarkers that increase during mastitis in buffaloes.

## 4. Endometritis

Endometritis is the most common uterine lesion observed in buffaloes slaughtered at abattoirs [76], mostly originating from postpartum uterine contamination by non-specific pathogens, which are usually eliminated in the process of involution. However, during the peripartum period, animals can undergo pronounced physiological changes that suppress both the cellular and humoral defense mechanisms by modulating the PMN phagocytic function and gene expression of the hosts [77], thus increasing animals’ susceptibility to various uterine infections [78], among which is endometritis [79]. The infertility associated with endometritis is correlated with tissue damage and inflammation [80]. During endometritis infection, the TLRs present on endometrial cells with CD14 recognize the pathogens associated with DNA, lipid, and LPS, stimulating the cytokine cascade with the release of pro-inflammatory cytokines, including TNF-α, IL-1, IL-6, and IL-8 [81] and a concomitant increase in the AMP expression of endometrial cells [82]. Specifically, chemokines attract PMNs and macrophages to eliminate the bacteria by increasing the generation of reactive oxygen species (ROS), and the persistence of PMNs in the endometrium in the absence of bacteria represents the primary characteristic of sub-clinical endometritis [83]. It is shown that peripheral PMNs isolated from endometritic cattle have lower phagocytic activity; therefore, it can be affirmed that the capacity of PMNs to eliminate pathogens is compromised during endometritis [82]. Different routine methods are used to diagnose clinical endometritis (CE), such as the rectal examination [84], vaginoscopic examination [85], endometritis clinical score [86], white side test [87], and uterine biopsy [88]. On the contrary, diagnosis of a sub-clinical (SCE) condition is based mainly on histological findings of endometrial tissue and the number of PMNs in uterine cytology, although the percentage of PMN cells diagnostic for SCE is variable [89]. Studies on the occurrence of the CE rate have reported an incidence ranging from 13.8% [76,90] to 22.4%, 24.7%, and 25% [91,92] in Iraqi, Iranian, and Egyptian buffalo cows, respectively. On the contrary, more discrepancy has been found regarding the occurrence rate of SCE, which can be ascribed to the health condition of buffaloes after calving and ranges from approximately 16.6% [90] to 20% [93] in Iraqi and Egyptian buffalo cows. Therefore, in buffaloes, the early diagnosis of SCE is an emerging issue that needs to be elucidated. In this regard, in the first comprehensive report of Loyi et al. [94], the mRNA expression of buffalo endometrial cytokine with CE and SCE was measured, and a high expression of CD14, TLR4, IL1β, IL6, IL8, and TNF-*α* in the endometrium of buffaloes with CE was found. Moreover, a significant up-regulation of CD14 (1 to 2-fold), IL-6 (15 to 36-fold), IL-8 (8 to 14-fold), and TNF-α (10 to 11-fold) mRNA has also been detected in SCE [94]. Accordingly, in a study of Bhadaniya et al. [95], a higher expression of CD14 and TLR-4 and of proinflammatory cytokines (IL-1β, IL-6, IL-8, and TNF-α) was found in the uterine tissue during the follicular phase and luteal phase in CE and SCE than in healthy buffaloes. An increase in the relative mRNA abundance of pro-inflammatory cytokines (IL-1β, IL-8, IL-6, and TNF-α) has also been detected in the cervical mucus of buffalo along with low intra-follicular estradiol levels, which suggests that endometritis could impede the follicular steroidogenesis [96]. In a study by Taha et al. [97], uterine tissue from buffalo suffering endometritis infection has expressed an upregulation of *IL-1α*, *IL-1β*, and *IL-6* genes, while *IL-10* and *TNF-α* genes have been down-regulated; this is the only paper in which the evaluation of the IL-10 gene expression during endometritis was evaluated. Therefore, the behavior of the IL-10 gene and its expression needs to be deeply explored in order to identify its role in endometritis-infected buffaloes.

In response to uterine infection, AAPs are synthesized, which have increased levels in serum through the first few weeks after calving [98,99]. Moreover, a high level of cytokines are effective stimulators to APPs such as CRP, haptoglobin, and serum amyloid A [100,101]. Both the upregulation of APPs and the PMN functions are key factors for the successful recovery from inflammation, for healthy uterine involution, and for obtaining a high conception rate [88]. Among the APPs, the C-reactive protein is an important element of the innate defense system [102], which encounters pathogenic bacteria and protects the tissues against further damage as well as helping in tissue regeneration and repair [100,103]. In a recent paper by Elsayed et al. [104], serum and endometrium showed higher values of TNF-α, IL-8, and serum CRP levels in buffalo suffering subclinical endometritis than in healthy buffalo; furthermore, a positive correlation among the percentage of endometrial PMNs and the serum and endometrial TNF-α, IL-8, and serum CRP has been found.

## 5. Climate Change-Induced Heat Stress

The heat stress condition is originated when a deviation in the ambient temperature upper critical thermoneutral zone is registered, and animals need more energy to lose body heat to maintain core body temperature above maintenance requirements and to achieve homeostasis [105]. An exhaustive review of the thermoregulatory response activated by buffaloes during heat stress exposition has been recently summarized by many authors [1,106,107]. Various complex coordinated mechanisms along with behavioral, physiological, neuro-endocrine, and molecular responses determine the success of the heat adaptability of buffalo. Moreover, both the neuro-endocrine (i.e., cortisol and thyroid hormone) and the molecular responses (i.e., heat shock proteins and circulating microRNAs) could have a direct effect on immune response under heat stress exposition. Indeed, it has been demonstrated that high levels of blood cortisol negatively affect the immune status of the Murrah buffalo calves during heat stress exposition [108]. In a recent comparative study between buffalo and bovine after an in vitro hyperthermia, buffaloes’ leukocytes have exhibited higher thermal adaptation compared to bovine cells, although in both species, the capacity of neutrophils to phagocyte has been decreased, which could be responsible for higher susceptibility to microbial infection under heat stress exposition [109]. Many studies have demonstrated that heat stress exposition impairs the lymphocyte function in buffaloes, reducing the cell-mediated immune responses [108,110,111]. The shift from a reduced cellular immunity versus promotion of the humoral immune response is mainly driven by the alteration of T-helper (Th)1/Th2 cytokine balance in favor of the secretion of Th2 (IL-4, IL-10, and IL-13) [112]. In addition, the cellular response to heat stress involves the synthesis of a subgroup of molecular chaperones named heat shock proteins (HSPs), among which the HSP70 can also stimulate cytokine production [113,114]. A study conducted in buffaloes exposed to 45 °C in a climatic chamber showed a higher serum concentration of HSP70 and a concomitant reduction in both the lymphocyte proliferative response and IL-2 secretion was found [115]. In a recent study by Ciliberti et al. [116], buffaloes were subjected to three temperature–humidity index (THI) classes as a measure of heat stress exposure; it has been demonstrated that the cytokine profile of serum milk can be useful in assessing the immune competence of dairy buffaloes, showing a transient Th2-type response due to a high level of IL-10, IL-4, and IFN-γ in the highest THI class. In addition, the myeloperoxidase activity of SC extracts and the expression of MPO on the surface of SCs were measured, demonstrating the absence of an inflammatory state in buffaloes subjected to heat stress, which has also been confirmed by the results of differential somatic cell counts.

In Pawar et al. [117], the increase in the proinflammatory cytokines IL-12, TNF-α, granulocyte-macrophage colony-stimulating factor (GMCSF), higher HSP70, and heat shock transcription factor 1 (HSF1) expression were show during heat or cold stress exposition; moreover, a negative correlation between milk production and both HSP70 and HSF1 was reported. In the mentioned study, the proportionate increase in IL-12, TNF-α, and GMCSF can indicate a cytoprotective role of HSP70 against stressors. In another study by Mishra et al. [108], an increase in serum HSP70 concentrations with a modulation of the immune response during heat stress exposure was reported. In Mukherjee et al. [118] study, the concentration of HSP70 was increased both in in vivo as well as in vitro lymphocytes under heat stress conditions. The results from the previous studies underline that in buffalo, the exposition of lymphocytes to in vitro high temperatures results in a depression of cell-mediated immunity response mediated by a high level of HSP70 and a reduction in proinflammatory cytokines. The impairment of immune function, which occurs under heat stress exposition [119], could also be caused by oxidative damage [120]. During inflammatory reactions, neutrophils and macrophages are activated to produce enzymatically, via NADPH oxidase, the anion superoxide radical (O^2•−^), which is the major ROS produced within the mitochondria [121]. However, when the production of ROS exceeds the capacity of antioxidant defenses to neutralize them, an oxidative stress condition occurs [122,123]. This condition is confirmed in animals exposed to ROS induced by heat, which in turn can lead to cytotoxicity [124]. Accordingly, in studies conducted on buffaloes during the summer season, higher levels of oxidative biomarkers [116,125] and lower levels of plasma antioxidant enzymes [126] have been registered. Consequently, buffaloes during the hot season have expressed a reduced total antioxidant capacity, which is not enough to cope with the excessive ROS produced by heat. This statement is confirmed by a study in which a dietary supplementation based on salt (sodium bicarbonate, potassium carbonate) and ascorbic acid in buffaloes exposed to thermal stress partially restored the antioxidant status of erythrocytes; however, higher lipid peroxidation was registered [12]. The controversial results on the oxidative status of buffalo under heat stress can be explained by the different methods and biomarkers utilized to measure it. Indeed, when the Oxidative Stress index (OSi) is calculated by combining both d-ROMs and BAP tests, a higher value of OSi during the cold rather than summer season in buffaloes was registered [127], demonstrating that heat stress is able to counterbalance the increase in ROS with a more active antioxidant system in a better way. In a study by Lan et al. [119], buffaloes exposed to oxidative stress mediated by both hypoxia (high altitude rearing condition) and heat stress exposition show a similar proteomic pattern of adaptation, which includes an enhancement of immunological proteomes, increasing levels of serum antioxidants, and inhibition of lipid oxidation [119].

## 6. Zoonoses and Other Disease

### 6.1. Fascioliasis

Fascioliasis is a zoonosis caused by *Fasciola hepatica* (*F. hepatica*) or *Fasciola gigantica* (*F. gigantica*) helminthic species [128], and represents a severe public health concern considering that about 2.4–17 million people become infected with *Fasciola* spp. [129]. In buffaloes [12,130], a mixed Th1 and Th2 immune response was implicated in the pathogenesis of *F. gigantica* infection. Particularly, *F. gigantica* modulates the buffalo host immune response [131] by induction of the Th2-related cytokines IL-6 and IL-8, suppression of Th1-cellular immunity [130,131], and downregulation of the major histocompatibility complex (MHC)-II-related genes [132]. The immune response activated by helminth also encompasses a strong correlation between Th1/Th17 [133]; thus, the regulation of IL-17 is critical to limit the inflammatory damage associated with *F. gigantica* infection. The immune response activated by helminths also encompasses IL-17, which is critical to limit the inflammatory damage associated with *F. gigantica* infection; therefore, a strong correlation between Th1/Th17 has been reported [133]. In this context, a paper by Zhang et al. [132] show a complex interplay among Th1, Th2, and Th17 controlled by high levels of IL-4, IL-10, and IL-13 cytokines and reduced levels of IFN-γ, IL-2, IL-6, IL-12, IL-17, and IL-1β cytokines during *F. gigantica* infection. Indeed, during the late phase of the infection, a state of immunosuppression was demonstrated with the promotion of Th2 and downregulation of the Th1/Th17-type inflammatory responses. An overall understanding of the specific immune response to *Fascicola* spp. is critical for the development of effective vaccines as well as the accurate diagnosis and treatment of fascioliasis [134]. In a recent study by Guo and Guo [135], 121 circulating host miRNAs were dysregulated in the serum of buffalo infected with *F. gigantica*, resulting in a high expression level of bta-miR-21-5p and bta-miR-23a and a gradual reduction in the bta-miR-125a expression level with the extension of *F. gigantica* infection time. In this paper, two parasite-derived circulating miRNAs (fgi-miR-87 and fgi-miR-71) have also been detected that may have diagnostic value in the early detection of *F. gigantica* infection in buffalo.

### 6.2. Bovine Viral Diarrhea Virus

Bovine Viral Diarrhea virus (BVDV) is a disease caused by a virus from the Pestivirus genus, family Flaviviridae [136], and can develop many different variants, namely type 1 (BVDV-1) and type 2 (BVDV-2), and a third putative bovine species, referred to as HoBi-like pestivirus [137]. The main characteristic of BVDV is the activation of immune response without a clear sign of infection; in this way, the virus continues to persist and replicate in infected animals and is released into the environment despite the presence of antibody and cell-mediated immunity. The mechanism that permits the virus to bypass the adaptive immune system by establishing tolerance remains unknown. Only a few studies have been conducted on the characterization of BVDV pathophysiology in buffaloes, with a special focus on immune responses. Some studies ascertain the presence of the BVDV-1 RNA in the fetal serum of buffaloes with mucosal disease [138,139] and the sub-genotype BVDV-1a [140] and BVDV-1b [141] viruses in aborted fetuses. In a study by Grandoni et al. [142], leukocyte subset distribution was performed during BVDV acute infection using a flow cytometric analysis. A transient leukopenia was found due to the reduction in all CD3^+^, CD4^+^, and CD8^+^ T lymphocytes, CD21^+^ B lymphocytes, and NK cells at 3- and 4-days post-infection without any variation in the CD4:CD8 ratio and in the number of B lymphocytes. The results obtained with the flow cytometric approach could be very informative for the diagnosis and/or prognosis of some pathological conditions that are asymptomatic, including BVDV [142]. The literature does not univocally indicate a Th1- or Th2-biased immune response in buffalo infected with BVDV despite the fact that balance seems to be necessary for the efficient elimination of an infectious agent and control of collateral damage. In a study by Mingala et al. [143], the cytokine expression of BVDV-infected buffaloes was performed, resulting in an up-regulation of TNF-α, IL-2, and IL-4 expressions and a down-regulation of IFN-γ and IL-12p40 expression. Moreover, in this study, swamp-type buffalo were considered more disease tolerant to BVDV than riverine-type buffalo, registering higher expression of both proinflammatory IFN-γ and TNF-α cytokines after an in vitro stimulation of PBMC with Concanavalin A.

### 6.3. Bovine Tuberculosis and Brucellosis

Bovine tuberculosis (BTB) is caused by a *Mycobacterium tuberculosis* complex infection, primarily *M. bovis* and *M. caprae*, and affects livestock species, wild animals, and humans (World Health Organization) [144]. The TB susceptibility of the buffalo [145,146,147] has garnered attention in areas where buffalo breeding is highly developed. *M. bovis* can be transmitted between animals firstly by inhalation and, to a lesser degree, by ingestion [148,149]. After inhalation, bacilli adhere to the alveolar surface of the lung and at this stage, macrophages can phagocytose them. Therefore, macrophages function as antigen-presenting cells via processing the mycobacterial antigens and presenting them to T-lymphocytes. In buffalo, lesions due to the tuberculosis infection are often localized in the lungs and lymph nodes but can also be found in other sites [150]. The principal studies performed on buffaloes are related to finding candidate genes for BTB susceptibility loci. For example, the IFNG locus codes for an immune signaling molecule named IFN-γ (Th1 cell responsiveness), which is essential for the host in eliminating *M. bovis* infection [151]. Indeed, IFN-γ is produced during adaptive immunity by T cells [22] and NK in response to exposure to bacterial antigens and has immunomodulatory properties [23], especially in naive animals (i.e., animals having no previous experience of a determined antigen). Therefore, the variability in the IFN-γ phenotype may be associated with fitness in facing the infection and consequently, with susceptibility to BTB [152]. Actually, BTB diagnosis in buffalo relies on IFN-γ-based tests that measure the strength of an individual’s immune response to *M. bovis* antigen challenge [153,154,155]; thus, culling based on variation in this response could drive selection based on the IFNG locus. Therefore, many investigations have been conducted in order to optimize the early diagnosis of BTB and eradicate this disease, mainly based on IFN-γ release and another panel of cytokines, as summarized in Table 2.

Apart from early diagnosis, another strategy is to apply breeding program based on genetic markers identifying BT resistant animals, such as gene polymorphisms associated with BT susceptibility. In a study of le Roex et al. [166], three genetic polymorphisms have been identified in the *SLC7A13*, *IL1α* and *DMBT1* genes that could have potential in the marker-assisted breeding program. Among the identified genes, both *DMBT1* and *IL-1α* genes exert an active role in mounting an effective immune response during infection including *M. bovis*. In another study, a novel mutation in bubaline *TLR2*, *TLR4* and *TLR9* genes, which are crucial in determining genetic disease resistance against *M. bovis*, have been recognized [167].

Brucellosis is one of the common zoonotic diseases caused by *Brucella abortus* bacteria, which triggers abortion, retention of placenta, and reduced productive and reproductive efficiency in natural hosts [168]. They are the main steps of *Brucella* infection: the first step is characterized by pathogen host invasion (within 2 days from infection), in the second step, which represents the acute phase of infection, the pathogen starts to replicate within different organs of the reticulo-endothelial and reproductive systems (2 days to 3 weeks from infection), and finally in the third step, if not properly treated, the brucellosis develops into a chronic infection in which the pathogen displays differences in the pathology of various tissues and lasts from up to 6 months to 1 year or more [169,170]. *Brucella abortus*, being an intracellular organism, elicits both the humoral and cell-mediated immune response throughout a cascade immune response activated by macrophage-derived cytokines such as IL-12 and TNF-α in the early responses to *Brucella* infection that led to the differentiation of naïve T cells into Th1 cells [171]. Subsequently, the activated Th1 cells produces IFN-γ and TNF-α that recruit phagocytes to the site of infection and boost the cytotoxic activity of NK cells and CD8+ cytotoxic T lymphocytes [172]. *Brucella* is able to activate an incredible and complex “stealth” strategy, generating a wrong “bar code”, which deflects any attempt of the innate and adaptive immune response to a prompt response to pathogen invasion [172] and actually helps this pathogen be transported into lymphoid tissues [173]. Therefore, in light of the recent high incidence of brucellosis in buffalo farms and the incredible resistance of *Brucella* to immune protective mechanisms activated by the host, the most appropriate method for the control of this disease is the immunization of susceptible animals [174]. With this in mind, *B. abortus* S19 is the most widely used vaccine for the prevention of bovine brucellosis. The first studies on the effectiveness of the *B. abortus* S 19 vaccine in buffalo calves and heifers demonstrated the activation of a good humoral immune response [168,175]. In a more recent study by Shome et al. [171], a reduced dose of the S19 vaccine to determine the effective immunizing dose in water buffaloes was tested. The results have demonstrated that the full dose (40 × 10^9^ CFU/dose) together with the 1/10th and 1/20th reduced doses are able to elicit the pathogen-specific antibody response and induce the secretion of IL-12, TNF-α, and IFN-γ with CD4+ and CD8+ effector T cell responses. This evidence is of particular interest because a reduction in the antibody titer could reduce the number of false positives and, at the same time, will cover a larger number of vaccinated animals. Another key point to the timely diagnosis of brucellosis is the combination of serological tests (ELISA, complement fixation test, serum agglutination test, or milk ring test), based on the detection of antibodies produced in response to *Brucella* infection, with bacterial detection methods, such as bacterial culture or molecular techniques [176]. Indeed, serological tests cannot be specific due to some limits such as the cross-reactivity reaction with other common antigenically related bacteria [177,178] and the absence of discrimination between seropositive animals that could be represented by individuals that have been infected and have recovered, or resistant animals that have simply been exposed to *Brucella*, as demonstrated also in buffalo [176]. Another commercially available vaccine against bovine brucellosis is the RB51; however, the antibodies induced by RB51 do not react in standard agglutination tests (Rose Bengal test (RBT), the serum agglutination test (SAT), or complement fixation test (CFT) [179,180]. Additionally, even if the RB51 vaccine may be safe and effective in preventing abortion and managing the spread of *Brucella* spp. in cattle [181], no international guidelines or protocols exist for its use in water buffaloes [182]. More recently, the efficacy of the RB51 vaccine in protecting or preventing/reducing the prevalence of this disease in buffalo farms was demonstrated [183]. However, the authors declare the limitations of their study conducted in Iran under specific climates, farming practices, and breeds of buffaloes, and therefore, the effectiveness of this vaccine needs to be further explored.

### 6.4. Bubaline Alphaherpesvirus-1 (BuAHV-1)

Bovine alphaherpesvirus 1 (BoHV-1) and Bubaline alphaherpesvirus 1 (BuHV-1) are pathogens that cause infection in buffalo [184] that are mainly characterized by an asymptomatic pattern in water buffalo adults, while buffalo calves are affected by slight respiratory and general illness symptoms (coughing, sneezing, wheezing, nasal secretions, fever, and loss of appetite) [185]. It has been described that both humoral and cell-mediated immune responses are involved during BoAHV-1 infection [186]; in the first stage, monocytes and dendritic cells from the innate immune responses are activated, and subsequently, the antigen is presented to CD4-positive T helper cells, which in turn start the polarization into different functional subsets (Th1, Th2, or Th17) [187,188]. Very few experiments have been conducted on the specific immune behavior to BuAHV-1 acted by buffalo. Recently, Grandoni et al. [189] defined the leucocyte subsets in both vaccinated and non-vaccinated water buffalo in response to experimental infection with BuAHV-1. In this study, after 15 days post-BuAHV-1 challenge, a condition of early leukopenia and lymphopenia has been found following neutrophilia and leukocytosis at 30 days post-challenge. Those results confirm the immune phenotyping of leukopenia and lymphopenia during BuAHV-1 is associated also with BoAHV-1 infection [190,191]. Interestingly, an increase in the intermediate monocyte (intMs) absolute number was registered in both the control and vaccinated groups. This result supports the important role of these cells, which gained attention in both human [192,193] and bovine [194] studies that are specialized in regulatory/anti-inflammatory functions and tissue repair and in antiviral responses and T cell immunomodulation. In another study, Lecchi et al. [195] characterized the miRNA profile of nasal secretion produced by the host and BuHV-1. Among the miR-148a-3p and miR-370-3p differentially expressed by the host, only the miR-370-3p was found capable of discriminating vaccinated and control animals with high sensitivity and specificity. Thus, miR-370-3p can be considered a biomarker and a candidate for an in-field and rapid screening of BuHV-1 [195]. Moreover, gene ontology analysis of miRNAs implicated during BuHV-1 infection has revealed modulation of genes involved in the cell adhesion pathway of the neuronal and immune systems.

## 7. Conclusions

In this review, some major immune mechanisms during the exposure of buffaloes to infectious and non-infectious stressors were outlined. In the course of mastitis, buffaloes increase the expression of immune genes related to the activation of pathogen recognition (TRL-2, TRL-4) and to the cytokine network (TNF-α, IL-1β, and IL-8), leading to the secretion of pro-inflammatory cytokines. Endometritis is characterized by the high expression of CD14, TLR4, IL-1α, IL1-β, IL-6, IL-8, and TNF-α in uterine tissue and cervical mucus. Heat stress induced by climate change causes an immune depression measured by a decreased lymphoproliferative response; in addition, a high level of HSP70 and a complex cross-talk between pro- and anti-inflammatory cytokines and oxidative stress is reported. However, very few experiments have been conducted on the immune competence of buffaloes under conditions of climate change-induced heat stress. Also, during zoonoses, including Fascioliasis, Tuberculosis, and Brucellosis, knowledge of the immune response is considered crucial to designing effective therapy and defining proper strategies to protect animals from the consequences of distress.

Cytokines and miRNAs emerged as the main biomarkers monitored during both infectious and non-infectious stressors. However, the evaluation of such markers should also be implemented in different pivotal physiological stressors, such as post-partum and in the dry period, as well as in pathological stressors in order to gain deep and wide comprehension of their biological and therapeutic role in buffalo welfare.

## Figures and Tables

**Table 1:** Description of the immune biomarkers involved during mastitis in buffalo.

Mastitis Definition	Animals	Type of Sample	Immune Biomarkers	References
Subclinical mastitis	Water buffalo	Somatic cell	*TRL-2*, *TRL-4*, *TNF-α*, *IL-1β*, and *IL-8* genes	[45]
Coliform or endotoxin-induced mastitis	Water buffalo	Liver, muscle, heart, hypothalamus, kidney, spleen, mammary gland, testis, and lungs	IL-1β, IL-6, IL-8, and IL-12 mRNA	[41]
Subclinical and clinical mastitis	Water buffalo	Plasma	TNF-α, IL-6, IL-1β, and IFN-γ secretion	[51]
*Staphylococcus aureus* and non-aureus staphylococci subclinical mastitis	Water buffalo	Milk	Vimentin, cathelicidins, histones, S100, neutrophil granule proteins, haptoglobin, and lysozyme proteins	[53]
California Mastitis Test positive animals	Water buffalo	Somatic cell	*CSF3* gene	[67]
*Staphylococcus aureus*, *Streptococcus viridans*, and *E. coli*	-	Hystological examination	CD8+ lymphocytes infiltrate	[68]
*-*	Water buffalo	Data from the literature search	*CXCL8*, *CD14*, *S100A8*, *CSF2*, *C3*, *Interferon regulatory factor 1 (IRF1)*, *IL1B*, *IL6*, *LPO*, *LBP*, *TLR2*, and *TLR4* genes	[70]
California Mastitis Test positive animals	Water buffalo	Milk	miR-146a, and miR-383	[74]

- not available.

**Table 2:** Main criteria and methods to select positive animals for the early diagnosis of bovine tuberculosis in buffaloes.

Cytokines	Aim	Criteria for Positive TB	Methods	Animals	References
IFN-γ	Evaluation of Mabtech bovine IFN-γ ELISAPRO	Blood samples stimulated with the QuantiFERON^®^ TB Gold Plus antigen	ELISA	African buffaloes	[156]
IFN-γ	Development of IFN-γ test with combination of PPDs recombinant protein, a mixture of ESAT-6 and CFP-10 antigens and four different interpretative criteria.	Positive SITT and SICTT and/or a positive IFN-γ test confirmed by isolation of M. bovis in at least one animal.	ELISA	Water buffalo	[157]
IFN-γ	Investigation on the use of QFT tubes and the cattletype^®^ IFN-gamma ELISA, in a cattletype IFN-γ release assay (IGRA) to detect *M. bovis* infection	Positive SCITT buffaloes and positive Bovigam^®^ 1G IGRA buffaloes	ELISA	African buffaloes	[158]
IP-10 and IFN-γ	Parallel measurement of IFN-γ and IP-10 in QuantiFERON^®^-TB Gold (QFT) plasma	Positive SCITT, positive Bovigam^®^ IGRA [159,160], and QFT IGRA [158] and QFT IPRA [161]	ELISA	African buffaloes	[162]
IP-10, MIG, MCP-1, MCP-2, MCP-3 and IL1-RA	Evaluation of candidate biomarkers of cell-mediated immunity for the diagnosis of Mycobacterium bovis infection	Animals positive to SICTT [159] and mQFT assays [159,163,164]	ELISA	African buffaloes	[165]

Acronyms: Same-day initiation of tuberculosis treatment (SITT), Single Intradermal Comparative Tuberculin Test (SICTT); Interferon Gamma Release Assay (IGRA); QuantiFERON^®^-TB Gold (QFT); single comparative intradermal tuberculin test (SCITT); interferon gamma-inducible protein 10 release assay (IPRA); Purified protein derivative (PPD) Early secretory antigenic target 6 (ESAT-6); culture filtrate protein 10 (CFP-10); Interferon gamma-inducible protein 10 (IP-10), monokine induced by interferon-gamma (MIG), monocyte chemoattractant protein (MCP), interleukin 1 receptor antagonist (IL1-RA).

## Data Availability

Not applicable.
